# Identification of an APC Variant in a Patient with Clinical Attenuated Familial Adenomatous Polyposis

**DOI:** 10.1155/2014/432324

**Published:** 2014-03-27

**Authors:** Andrew T. Schlussel, Susan S. Donlon, Faye A. Eggerding, Ronald A. Gagliano

**Affiliations:** ^1^Department of General Surgery, Tripler Army Medical Center, 1 Jarrett White Road, Honolulu, HI 96859, USA; ^2^Huntington Medical Research Institutes, 99 N. El Molino Avenue, Pasadena, CA 91101-1830, USA

## Abstract

*Introduction.* The objective of this case report is to discuss an unclassified germline variant of the adenomatous polyposis coli (APC) gene identified in an older patient with attenuated familial adenomatous polyposis syndrome (AFAP).* Methods.* We present a case report of a 66-year-old man diagnosed with AFAP. Colonoscopy found multiple polyps and invasive adenocarcinoma arising in the transverse colon. Samples were tested for mutations in the APC gene.* Results.* DNA sequencing of germline DNA identified a cytosine (C) to thymine (T) transition at nucleotide 1240, heterozygous. The C to T transition at codon 414 is predicted to convert an arginine residue to a cysteine that is possibly pathogenic. Analysis of the patient's colon tumor DNA indicated that the tumor had lost the mutant variant allele and retained only the normal allele, suggesting that the variant may not be significant.* Conclusions.* The p.R414C variant has been described previously as a germline mutation of probable pathogenicity. This substitution should be considered an unclassified variant and possibly not pathogenic. These findings support the need for further genetic testing of tissue, as well as for developing a mechanism for testing all variants, as this could significantly impact the lives of patients and their family members.

## 1. Introduction

Familial adenomatous polyposis syndrome (FAP) is found in less than 1% of all colorectal cancers. Patients with this autosomal dominant syndrome will progress to colon cancer nearly 100% of the time [1]. Over 20 years ago, the adenomatous polyposis coli (APC) gene was identified and mutation of the APC gene, located on chromosome 5q21-22, was found to be the cause of FAP [2]. This tumor suppressor gene consists of 15 exons which encode a protein of 2843 amino acids. It functions in cellular processes, including transcription, cell cycle control, migration, differentiation, and apoptosis. More specifically, the APC gene encodes a protein that affects the Wnt signaling pathway, which is involved in signal transduction and degrading ß-catenin in the cell's cytoplasm [3]. Improper ß-catenin accumulation predisposes to development of colon tumors [4].

Somatic APC mutations are found in the majority of sporadic colorectal cancers [5]. However, in FAP families, the disease occurs as the result of the typical two-hit model of tumor suppressor dysregulation, where one mutation is inherited and the loss or mutation of the normal allele occurs as a somatic event during tumor development [2]. The majority of APC gene mutations are deletions or insertions or nonsense mutations which result in the formation of a truncated APC protein. APC gene mutations at codons 1061 and 1309 account for 11% and 17%, respectively, of all its germline mutations [1, 3]. The genotypic understanding of the APC mutation is clinically relevant to the phenotypic presentation. It is known that classic FAP occurs when there are mutations between codons 168 and 1580 with severe disease between codons 1250 and 1464 [6]. Classical FAP is defined clinically if there are one hundred or more adenomatous colon and rectal polyps and typically occurs in patients younger than forty. Alternatively, FAP can be diagnosed in a patient with fewer than 100 adenomatous polyps who has a relative with FAP [7]. These patients have a nearly 100% risk of progression to cancer by 60 years of age.

These genotype-phenotype correlations occur based on the location of the germline mutation on the APC gene. This concept is further elucidated in patients with attenuated FAP, where the genotypic mutation occurs in the 5′ (codons 78–167) and 3′ (codons 1581–3843) regions of the APC gene [3]. There is lack of data supporting a perfect algorithm for a diagnosis of attenuated FAP, but it is generally defined in individuals with 10–99 colonic adenomatous polyps, with 100 or more polyps occurring at an older age, a history of colorectal cancer before 60, and a family history of multiple adenomatous polyps [7, 8]. Although the presentation is very variable, most of these patients will have fewer than 100 polyps, sometimes even 4-5. Furthermore, these patients will usually have rectal-polyp sparing, have right-sided colonic adenomas, and usually lack extra colonic manifestations [2]. Because of the greater genotypic and phenotypic variability in attenuated FAP, a high clinical suspicion is critical for the diagnosis, as these patients frequently, if not always, progress to a colon cancer [2].

Although there have been multiple studies describing the sequence and the location of the APC gene, the significance of single amino acid missense variants in the APC gene is difficult to interpret [2]. With the advent of genetic testing, it has become important to characterize these variants in order to properly counsel and treat FAP patients, particularly those patients with the attenuated form of FAP. There are many mutations or polymorphisms that can occur in the APC gene; however, not all of these mutations will result in cancer. The purpose of this case report is to discuss the significance of a previously reported germline variant of the APC gene, p.R414C, in relation to the development of polyposis and colon cancer in a patient with a clinical diagnosis of attenuated FAP.

## 2. Case Report

This is a case of a 66-year-old man who presented with bright red blood per rectum for approximately two months. He has a past medical history significant for benign prostatic hypertrophy and obstructive sleep apnea, and his only surgery was a vasectomy. He reported being a social drinker and previously smoked but quit more than 30 years ago. The patient was adopted; however, he is aware of his biologic mother who had an unknown gynecological cancer resulting in death. He also has multiple maternal uncles who died from cancer of an unknown etiology with the earliest death between 30 and 40 years of age. His physical exam was normal. A recent colonoscopy demonstrated a 3 cm friable mass in the transverse colon at approximately 70 cm. There was a polyp in the cecum which measured 4 mm, polyps in the ascending colon measuring 1.5 cm and 4 mm, and a polyp in the sigmoid colon measuring 8 mm. Histologically, he had an invasive adenocarcinoma arising from a tubular adenoma in the transverse colon. There were findings of tubular adenomas with high grade dysplasia in the descending colon as well as tubular adenomas in the sigmoid and ascending colon. A carcinoembryonic antigen level was normal, and a CT scan of the chest, abdomen, and pelvis was only significant for circumferential wall thickening in the transverse colon. The patient underwent a subtotal abdominal colectomy, removing all lesions. The pathological evaluation identified moderately differentiated adenocarcinoma of the transverse colon with twenty-three benign lymph nodes.

Due to the patient's cancer in the transverse colon, with another synchronous high grade dysplastic lesion in the descending colon, and a maternal history of a young gynecologic cancer, genetic analysis of the patient's germline and tumor was performed. Results were obtained from the Molecular Oncology and Cancer Genetics Laboratory, Huntington Medical Research Institute, Pasadena, CA. DNA sequencing of the patient's germline identified a cytosine (C) to thymine (T) transition at codon 414, exon 9, and c.1240 C > T, heterozygous in one allele of the APC gene. The C to T transition at codon 414 is predicted to convert an arginine residue to a cysteine. This result was confirmed by analysis of duplicate samples and by sequencing in both directions. DNA sequence analysis identified a guanine (G) to adenine (A) transition at codon 2502 (c.7504 G > A, heterozygous). The G to A change at codon 2502 is predicted to convert a glycine to a serine residue; it is considered a polymorphism and the frequency of the variant p.2502S allele is estimated at 2.8% [9]. The final interpretation of the germline genetic analysis identified two variants: APC gene unclassified variant p.R414C; c.1240 C > T and APC polymorphism: p.G2502S (c.7504 G > A). The patient tested negative for MYH gene coding regions and for Lynch syndrome. To further elucidate the potential deleterious effects of this variant on the formation of FAP associated colorectal cancer, sequence analysis of the patient's adenomas and tumor tissue was performed. Analysis of the adenoma demonstrated the presence of the p.R414C variant; however, tumor tissue analysis demonstrated loss of heterozygosity. The tumor had lost the allele carrying the p.R414C variant and retained only the normal allele.

## 3. Discussion

Previous epidemiologic studies have shown that 15–30% of colorectal cancers have a hereditary component. Only 2–5% of colorectal cancers are the result of dominantly inherited cancer syndromes as FAP, MYH-associated polyposis, or hereditary nonpolyposis colon cancer (HNPCC or Lynch syndrome). The role of rare nonsynonymous missense variants of the APC gene in colorectal cancer predisposition is not clear [4]. Understanding the genes responsible for the progression of a benign adenoma to a colorectal neoplasia is important not only for diagnosis but also for the counseling of patients and their families. Genetic analysis has linked the gene or genes responsible for FAP to chromosome 5q21. Germline APC mutation is not a common cause of colorectal cancer, but somatic mutations of the APC gene occur in about 80% of sporadic colon cancers. Moreover, somatic mutations in the APC gene are an early event in colorectal tumorigenesis and in fact somatic mutations are present in adenomas less than 1 mm in size. Inactivation of both alleles of the APC gene is common in colon cancers.

The R414C mutation was first identified by Nishisho and colleagues in 1991 who were originally attempting to analyze four possible genes (MCC, TB2, SRP, and APC) which were involved in this inherited form of colon cancer in a cohort of ninety FAP patients. Through cloning, PCR, and DNA sequencing, they were able to identify a cytosine to thymine transition at codon 414 which resulted in a change from arginine to cysteine. Furthermore, they showed that this amino acid change was not seen in a sample of 200 individuals without FAP, inferring clinical significance. This study was important as it was one of the first studies to show that patients could be directly tested for variants in alleles associated with FAP [10].

The R414C APC gene variant was again encountered in 2008 by Azzopardi et al., in an attempt to identify the role of nonsynonymous variants in the APC gene sequenced in almost 700 patients with FAP and about 1,000 healthy matched controls. This study identified just over sixty variants that were overexpressed in patients that did not have conventional mutations in the APC gene. They were able to identify three FAP patients that carried the R414C variant. Overall, only about 10% of the FAP patients carried a rare nonsynonymous variant, but about 17% of non-FAP patients had a rare variant in the APC gene. The G2502S polymorphism but not the R414C variant was seen in the group of healthy control patients. This study was able to show the probable benign nature of the G2502S variant but inferred the probable clinical significance of the R414C variant [4].

Germline genetic analysis of our patient identified the p.R414C variant in one allele of the patient's APC gene, a finding consistent with the patient's clinical presentation. This rare variant has only been described in three previous papers and this was the first time it has been identified at the Huntington Medical Research Institute, Pasadena, CA, an academic facility with experience in molecular oncology and cancer genetics [2, 4, 8]. The loss of heterozygosity in this patient's tumor suggests either that the p.R414C mutation may not be of clinical significance or that it may be involved in the initiation of cancer development, but it is dispensable for the progression of cancer. The presence of the p.R414C allele in the DNA of the tubular adenomas supports this interpretation. Our analysis of the R414C variant demonstrates the potential clinical importance of thorough genetic analysis of patients with unclear inherited cancer conditions. Study of additional affected and unaffected family members is also helpful in cases of variants of uncertain significance. Moreover, the ability to analyze the tumor for loss of heterozygosity enhances the interpretation of the results. These results will not only improve medical knowledge and augment the genetic database, but these findings are also critical to the counseling and recommendations for further testing of patient families.

In conclusion, the p.R414C variant has been described previously as a germline mutation of probable pathogenicity [10]. Our studies suggest that the p.R414C substitution should be considered an unclassified variant and not pathogenic. These findings support the need for further genetic testing of tissue, as well as for developing a mechanism for testing all variants for functional activity, as this could significantly impact the lives of patients and their family members.
